# Laparoscopic cholecystectomy for triple gallbladder malformation: A comprehensive case report and literature review

**DOI:** 10.1097/MD.0000000000041190

**Published:** 2025-01-10

**Authors:** Shuang-hao Zhou, Zhen-hua Li, Yao-chen Wei, Zhi-yu Wu, Qing-jiang Fu, Li-ying Cao, Xiang-ming Ma

**Affiliations:** a Hepatobiliary Surgery, Kailuan General Hospital, Tangshan, Hebei Province, China; b Graduate School, North China University of Science and Technology, Tangshan, Hebei Province, China; c Hepatobiliary Surgery, Hepatobiliary Disease Laboratory, Kailuan General Hospital, Tangshan, Hebei Province, China.

**Keywords:** gallbladder malformation, laparoscopic cholecystectomy, magnetic resonance cholangiopancreatography (MRCP), triple gallbladder

## Abstract

**Rationale:**

Triple gallbladder is a rare congenital anatomical abnormality because of the incomplete regression of rudimentary bile ducts and is often not found until it is accidentally detected during imaging research.

**Patient concerns:**

We report a rare case of triple gallbladder malformation and review the English literature on biliary tract variation caused by gallbladder malformation. The diagnosis, treatment, and postoperative situation of the patients were summarized and analyzed.

**Diagnoses:**

The patient was diagnosed with triple gallbladder malformation.

**Interventions:**

We conducted laparoscopic cholecystectomy for the patient.

**Outcomes:**

We successfully removed all 3 gallbladders by laparoscopic cholecystectomy, and the patient had no postoperative complications such as bleeding or bile leakage and recovered well.

**Lessons:**

At present, the best treatment for triple gallbladder malformation is to remove all 3 gallbladders by laparoscopic cholecystectomy, which can effectively prevent the occurrence of “postcholecystectomy syndrome” caused by individual gallbladder residue. However, the diagnosis of triple gallbladder is very challenging and often requires a combination of advanced imaging methods. However, sometimes preoperative imaging does not fully reveal biliary tract variation. Therefore, we need to rely on the guidance of evidence-based medicine before surgery and the accurate evaluation of surgical plans to complete the operation without risk.

## 1. Introduction

A triple gallbladder malformation is an exceptionally rare congenital anomaly of the biliary system.^[1]^ Thus far, according to the global literature, only 21 cases have been documented, with laparoscopic removal of the triple gallbladder performed in only 5 instances. We recently found the 22nd case of triple gallbladder abnormality and successfully performed laparoscopic cholecystectomy, making it the 6th recorded case of its kind.^[2]^

Although preoperative magnetic resonance cholangiopancreatography (MRCP) is important for the diagnosis of gallbladder malformations,^[2]^ it does not provide a definitive diagnosis in this extremely rare case. Careful dissection of the biliary tract during surgery allows the identification of triple gallbladder malformations. Subsequently, we performed laparoscopic removal of all 3 gallbladders. After surgery, we conducted a detailed review of existing reported cases of triple gallbladder malformations to enhance our understanding of triple gallbladder malformations.

## 2. Case presentation

We report the case of a Chinese Han female who visited our hospital with cholelithiasis and gallbladder space-occupying lesions that were found during physical examination. She had a 2-month history of hypertension, and her blood pressure was controlled at 140/90 mm Hg. No history of genetic disorders. Physical examination revealed a flat abdomen with no gastrointestinal pattern or peristaltic waves. Varicose veins were not observed in the abdomen. The abdomen was soft to palpation with no palpable mass and there was no pressure, rebound pain or muscle tension throughout the abdomen. No liver or spleen was palpated and Murphy’s sign was negative.

Enhanced epigastric computed tomography indicated cholecystolithiasis and cholecystitis, and epigastric magnetic resonance imaging and nuclear magnetic hydrography of the bile duct indicated double gallbladder type I with multiple cholecystolithiasis and a low bile duct.

The complete blood count and chemistry were unremarkable. Based on the preoperative examination results, we concluded that the patient had double gallbladder malformation. After communication between the patient and her family, we decided to perform a laparoscopic double cholecystectomy. To better understand the structure of the biliary tract during surgery, we performed a 3D laparoscopy. Before surgery, we carefully observed the MRCP images of the patient, considering that the patient had a double gallbladder malformation and a low confluent gallbladder duct. In addition, we carefully searched PubMed before surgery and applied UpToDate to fully understand the situation of double gallbladder malformations and planned various scenarios that may occur during the operation to be able to perform surgery more accurately.

During the surgery, the liver was normal in size and shape, with no tumors or nodules. Two gallbladder attachments were observed on the right liver surface. The larger gallbladder was located on the right side, approximately 7 × 4 cm, and the smaller gallbladder was located on the left side, approximately 5 × 2 cm. It was grayish-white in color, without obvious edema, and was slightly adherent to the surrounding omentum. No obvious abnormalities were observed in any other structures.

Thus far, we still think that it is a double gallbladder malformation. Double gallbladder malformations significantly increase the anatomical complexity of the gallbladder triangles. Therefore, we did not blindly remove the gallbladder duct, but carefully freed the gallbladder duct first and did not treat the gallbladder duct. Instead, retrograde resection was performed; the right gallbladder was first separated retrograde and free to the gallbladder duct; the left gallbladder was separated retrograde, and a small cystic structure, approximately 2 × 1 cm in size, was observed between Hartmann’s pouch of the 2 gallbladders. We repeatedly observed this cystic structure and confirmed that the patient had triple gallbladder malformation.

**Figure 1.**
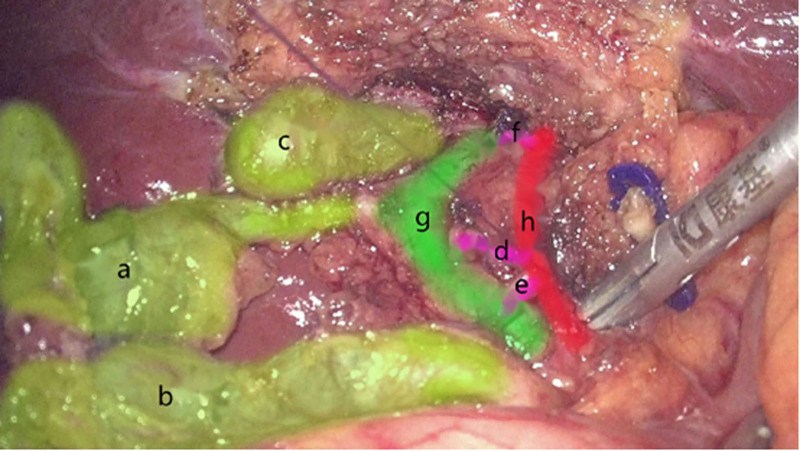
Three gallbladders and their corresponding cystic arteries(A) gallbladder 1; (B) gallbladder 2; (C) gallbladder 3; (D) arteries of gallbladder 1; (E) arteries of gallbladder 2; (F) arteries of gallbladder 3; (G) common bile duct; (H) hepatic artery.

We carefully freed 3 different sizes of the gallbladder and found that the left gallbladder duct down to the duodenum into the common bile duct, the cholecystic duct of the right gallbladder normally flows into the common bile duct, the gallbladder duct flows into the right hepatic duct, and the 3 gallbladder have their own independent gallbladder blood supplies (Fig. 1). Each gallbladder duct was clamped with a biological clamp 0.5 cm away from each gallbladder merging into the common bile duct. The proximal end of the 3 gallbladder ducts and the corresponding gallbladder arteries were sewn with PDS-II sutures, and each artery was excised. Each gallbladder duct was cut between the biological clamp and suture and the wound was stopped by electrocoagulation. After checking for no active bleeding or bile leakage, 3 gallbladders were removed from the subxiphoid incision using plastic bags. After checking the instruments and gauze, a hemostatic gauze was placed in the operating area, anti-adhesion sodium hyaluronate was sprayed into the abdominal cavity of the patient, and a minor omentum pore drainage tube was placed to drain abdominal fluid and release CO_2_ gas. Suture the incision and complete the procedure. The operating time was 105 minutes. No postoperative complications were noted. The patient attempted to drink water 6 hours after surgery. On the first day after the surgery, a small amount of non-greasy food was consumed several times. On the second day after surgery, there was no bleeding, bile leakage, or other complication. The sutures and drainage tubes were removed. On the morning of the third day after surgery, the patient was in good condition with no other physical discomfort and was allowed to leave the hospital.

## 3. Discussion

During embryonic development, the liver, gallbladder, and pancreas originate from the endoderm, and after 4 weeks of gestation, they originate from the foregut. It is anatomically divided into 2 parts: the hepatic head and the cystic tail. The gallbladder primordium initiates its formation at the distal extremity of the biliary duct during the intricate period of the 5th and 6th weeks of gestation, and any perturbation or deviation occurring during this critical juncture can culminate in an anomalous gallbladder or bile duct configuration, manifesting as malformations.^[3]^ Duplication of the gallbladder occurs due to division of the gallbladder primordium or excess primordium. The precise positioning of the gallbladder primordium during its developmental stages governs its distinct phenotype. When it arises in proximity to the common bile duct or hepatic duct, it undergoes differentiation to form an autonomous duct exclusively dedicated to the gallbladder, presenting a striking manifestation of its intricate and elegant embryonic transformation.^[3,4]^ Conversely, if a cholecystic progenitor develops within a pre-existing bile duct, it forms a shared bile duct for multiple gallbladders.^[5]^

**Figure 2.**
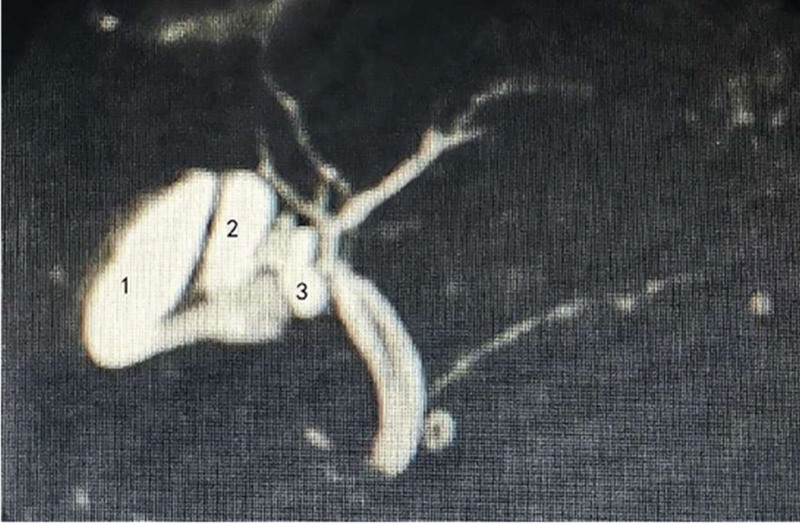
Three completely separate cystic structures.

A duplicate gallbladder malformation is an extremely rare congenital anomaly with clinical manifestations in only a minority of patients. The incidence of double gallbladder malformations found during cholecystectomy is approximately 1/12,000, whereas triple gallbladder malformations are extremely rare, with only 21 reported cases.^[3,6]^ After a careful literature search of triple gallbladder malformations in PubMed and Medline, we found that Alicioglu proposed a classification system for triple gallbladders based on the number and arrangement of gallbladder ducts that merge with the common bile duct. These classifications include: fusion of all 3 gallbladder ducts into a single duct before joining the common bile duct; fusion of 2 gallbladder ducts with each other prior to their entry into the common bile duct, while the third duct remains solely connected to the common bile duct; and separate entry of all 3 gallbladder ducts into the common bile duct.^[7]^ However, according to the alignment of the cystic duct and common bile duct in our case of triple gallbladder malformation, it did not follow the distribution of one of these 3 types as stated by Alicioglu, so we found a new way of distribution of the cystic duct and common bile duct in 3 gallbladders. In addition, although it has been documented that MRCP has a high diagnostic rate of about 97% for gallbladder malformations,^[8]^ as to why 3 gallbladder malformations were not detected on MRCP preoperatively, we considered the possible reason to be the presence of overlap of the gallbladder, cystic duct, and common bile duct. Postoperatively, we again viewed and analyzed the patient’s MRCP from multiple perspectives and eventually found a triple gallbladder abnormality (Fig. 2).

## 4. Conclusions

Patients with triple gallbladder malformations are highly prone to gallstones, cholecystitis, and gallbladder cancer. Currently, laparoscopic cholecystectomy and total cholecystectomy are considered the best treatment methods for triple gallbladder malformation, effectively preventing the occurrence of “post cholecystectomy syndrome” related to individual gallbladder preservation. It is necessary to thoroughly determine the anatomical relationship during surgery to prevent potential damage to the biliary system.

Anatomical variations of the gallbladder are best identified through imaging, especially when there are 3 gallbladders. Modern imaging technology can comprehensively evaluate biliary structures for accurate preoperative diagnosis, promote precise surgical planning, and minimize intraoperative risks. However, the diagnosis of triple gallbladder is very challenging and often requires a combination of advanced imaging methods, such as MRCP, to accurately define complex biliary structures. However, preoperative imaging cannot fully reveal biliary tract variations. In this case, preoperative MRCP does not accurately indicate the type of biliary confluence triple gallbladder malformation, but relies on a preoperative search for evidence-based medicine and precise evaluation of the surgical plan to complete the surgery with targeted and risk-free outcomes.

## Acknowledgments

Thanks to the 2024 medical science research project plan of Hebei Province (20242220).

## Author contributions

**Investigation:** Zhi-yu Wu.

**Methodology:** Yao-chen Wei.

**Resources:** Qing-jiang Fu.

**Supervision:** Li-ying Cao.

**Visualization:** Zhen-hua Li.

**Writing – original draft:** Shuang-hao Zhou.

**Writing – review & editing:** Xiang-ming Ma.
